# Mechanisms of action and applications of *Polygonatum sibiricum* polysaccharide at the intestinal mucosa barrier: a review

**DOI:** 10.3389/fphar.2024.1421607

**Published:** 2024-08-19

**Authors:** Yu Ren, Yi Sun, Yu-Ying Liao, Si Wang, Qian Liu, Chun-Yan Duan, Lan Sun, Xiao-Ya Li, Jia-Li Yuan

**Affiliations:** ^1^ Yunnan Provincial Key Laboratory of Integrated Traditional Chinese and Western Medicine for Chronic Diseasein Prevention and Treatment, School of Basic Medical Sciences, Yunnan University of Chinese Medicine, Kunming, Yunnan, China; ^2^ Xiyuan Hospital, China Academy of Chinese Medical Sciences, Beijing, China; ^3^ College of Traditional Chinese Medicine, Yunnan University of Chinese Medicine, Kunming, Yunnan, China; ^4^ First Clinical Medical College, Yunnan University of Chinese Medicine, Kunming, Yunnan, China

**Keywords:** *Polygonatum sibiricum*, *Polygonatum sibiricum* polysaccharide, homology of medicine and food, intestinal mucosal barrier, short-chain fatty acids

## Abstract

As a medicinal and edible homologous Chinese herb, *Polygonatum sibiricum* has been used as a primary ingredient in various functional and medicinal products. Damage to the intestinal mucosal barrier can lead to or worsen conditions such as type 2 diabetes and Alzheimer’s disease. Traditional Chinese medicine and its bioactive components can help prevent and manage these conditions by restoring the integrity of the intestinal mucosal barrier. This review delves into the mode of action of *P. sibiricum* polysaccharide in disease prevention and management through the restoration of the intestinal barrier. Polysaccharide from *P. sibiricum* effectively treats conditions by repairing the intestinal mucosal barrier, offering insights for treating complex diseases and supporting the application of *P. sibiricum* in clinical settings.

## 1 Introduction

*Polygonum sibiricum* (PS; in Chinese: Huang Jing) is the dry rhizome of *Polygonum kingianum* (PK; in Chinese: Dian Huang Jing or Yunnan Huang Jing) and *Polygonum cyrtonema* (PC; in Chinese: Duohua Huang Jing). PS is a member of the lily family. In traditional Chinese medicine (TCM), PS has the effects of replenishing qi and nourishing yin, strengthening the spleen, moistening the lung, and tonifying the kidney (Chen et al., 2021). In 2002, the Chinese Ministry of Health included PS in the list of substances that are both food and medicine. *Polygonatum sibiricum* polysaccharide (PSP) is the main active ingredient in PS (Liu S. et al., 2022). PSP has been reported to have many pharmacological applications and biological activities such as antioxidant activity and anti-aging activity. In addition, it has been shown to enhance immunity in immunosuppressed mice by improving gut microbes and activating macrophages (He et al., 2022).

The intestinal mucosal barrier is a multilayered tissue structure with specific functions and is the surface on which the organism interacts with the external environment (Farhadi et al., 2003). In recent years, the intestinal tract has become a research hotspot in the field of TCM, and the intestinal mucosal barrier is an important target for the action of most TCM and the treatment of diseases (Che et al., 2022). The intestinal mucosal barrier is the first line of defense against infiltration of luminal contents and performs many biological functions (Wu et al., 2023). It not only serves as a site for the absorption of food nutrients and microbial metabolites, but also acts as a barrier to prevent microbial invasion of the tissues and moderates the inflammatory response to the myriad of contents in the intestinal lumen. In addition, the intestinal mucosal barrier is plays an important role in immune defenses and in the maintenance of the integrity of the intestinal mucosa. According to a study published in *Carbohydr Polymers*, PSP can repair the intestinal mucosal barrier (Gong et al., 2024). In view of this, this paper will summarize the mechanism of PSP in repairing the intestinal mucosal barrier for the treatment of diseases, with a view to providing ideas for the clinical treatment of multisystemic diseases so as to provide a theoretical background for the development and clinical application of PS.

## 2 PS and PSP

### 2.1 PS

The name “Huangjing” derives from the traditional Chinese theory of “absorbing the essence of heaven and earth” (Zhao et al., 2018). Modern pharmacological studies have shown that PS has anticancer, anti-aging, blood sugar regulation, and anti-fatigue effects (Li X. L. et al., 2023). It is a medicinal and food herb, which has been developed into functional foods such as PS tea and PS liquor. PS is obtained from fruits or vegetables in daily life (Li X. L. et al., 2023), which replenishes the nutrients required for human growth and development (Xie et al., 2021), such as Zn, Ni, Pb, Sn, Mg, Mn, Fe, Li, Ca, glutamic acid, and aspartic acid. (Yao-Yao et al., 2022; Chen et al., 2024). PS has the potential to be developed into a novel and promising functional health food for older individuals. Studies have shown that PS can activate the BDNF/TrKB pathway to improve cognitive dysfunction and synaptic plasticity in naturally aging rats (Zhang et al., 2022). Furthermore, PS can effectively regulate glucose and lipid metabolism during the development of type 2 diabetes (T2D) as an alternative functional food (Wang G. et al., 2022). In the Chinese Pharmacopoeia, the clinical dosage of PS is 9–15 g. In current clinical records, the dosage of PS is mostly 12–15 g (Xu et al., 2024). More importantly, since ancient times, China has had a history of combining PS with other medicinal drugs to prevent and treat diseases, for example, Erjingwan during the Song dynasty. Erjingwan can reduce intestinal inflammatory reactions, restore intestinal mucosal barrier function, and reduce the level of neuroinflammatory factors by regulating the intestinal bacterial flora and participating in the metabolism of short-chain fatty acids (SCFA), thus representing a suitable treatment for Alzheimer’s disease (AD) (Li-ping et al., 2023). Most traditional Chinese medicines are taken orally, increasing the interactions between drugs and intestinal microorganisms, which may be the key to their efficacy. It is evident that targeting the regulation of intestinal microecology could be an important strategy for disease control (Chen and Houkai, 2020). Repairing the intestinal mucosal barrier by increasing the abundance of probiotics can alleviate the development of diseases such as functional dyspepsia,antibiotic-associated diarrhea and T2D (Medina-Vera et al., 2019; Mekonnen et al., 2020; Shen et al., 2024). In modern use, PS is often paired with other medicinal drugs to treat diseases. Yongwei (Fu Yong-wei, 2022) showed that *Polygonatum* and *Euryaleferox* powder composed of PS and *Semen Euryales*, *Dioscorea opposita Thunb*, *Semen ziziphi spinosae*, *Chinese-date*, *Radix codonopsis,* and *Patchouli* could significantly increase the abundance of *Bifidobacteria* and *Lactobacilli* in the intestinal tract, decrease that of *Escherichia coli* and *Enterococci*, and increase the body’s immune system and digestive and absorptive capacity, which in turn achieved an ameliorative effect on functional dyspepsia. Liu et al. (Liu Ying et al., 2023) showed that PS paired with *Polygonatum odoratum* and *Mulberry* leaf restored the intestinal mucosal barrier in rats with antibiotic-associated diarrhea by restoring the diversity and abundance of the intestinal flora, decreasing the content of pro-inflammatory inflammatory factors, alleviating inflammatory symptoms, and restoring the intestinal mucosal barrier. Song et al. (Song, 2023) showed that the compatibility of PS with *Pueraria lobata*, *Mulberry* leaf, *Poria cocos,* and *Lycium barbarum* can alleviate T2D by reducing systemic chronic low-grade inflammation caused by lipopolysaccharide (LPS) by regulating the proportion of Firmicutes and Bacteroides (F/B) in the intestinal flora (as shown in Table 1). Thus, the demand for PS has increased annually to 4,000 tons (Zhang J. et al., 2021).

**TABLE 1:** Mechanisms of PS in combination with other medicinal drugs for the treatment of diseases.

No.	Compatibility drugs	Function	Mechanisms of action	References
1	*Lycium chinense Miller*	Improvement of AD	Reduces intestinal inflammatory reactions, restores intestinal mucosal barrier function, and restores the level of neuroinflammatory factors by regulating intestinal bacterial flora and participating in the metabolism of SCFA	(Li-ping et al., 2023)
2	*Semen Euryales*, D*ioscorea opposita Thunb*, *Semen ziziphi spinosae*, *Chinese-date*, *Radix codonopsis* and *Patchouli*	Improve functional dyspepsia	Increases the number of *bifidobacteria* and *lactobacilli* in the intestinal tract, decreases the number of *Escherichia coli* and *Enterococci*, and increases the body’s immune system and digestive and absorptive capacity	(Fu Yong-wei, 2022)
3	*Polygonatum odoratum* and *Mulberry leaves*	Improvement of antibiotic-associated diarrhea	Restores the diversity and abundance of the intestinal flora, decreases the content of pro-inflammatory inflammatory factors, alleviating the inflammatory symptoms	(Liu Y. et al., 2023)
4	*Pueraria lobata*, *Mulberry* leaf, *Poria cocos*, *Lycium barbarum*	T2D mitigation	Reduces low-grade systemic inflammation caused by LPS by regulating the proportion of F/B in the intestinal flora	(Song, 2023)

### 2.2 PSP

The active constituents of PS include polysaccharides, steroidal saponins, and volatile oils, among which polysaccharides are the most important active constituents of PS. Polysaccharides are one of the four basic substances that constitute life, consisting of at least 10 or more monosaccharide molecules through condensation, and with the loss of water molecules forms polymers, the molecular structural formula is (C_6_H_10_O_5_)n and is widely found in nature, mainly in plant cell walls, animal cell membranes, and microbial cell walls (Xu et al., 2019). Polysaccharide content ranges from 11% to 22% and comprises five components, namely PSW1B-b, PSW2A-1, PSW3A-1, PSW4A, and PSW5B (Liu S. et al., 2022). The main distribution of the relative molecular masses of homogeneous polysaccharides isolated from PS to date ranges from 1.80 × 10^3^ to 6.28 × 10^5^ and predominantly consists of galactose (Gal) and mannose (Man), but also includes fructose (Fru), glucose (Glc), arabinose (Ara), and rhamnose (Rha), as well as small amounts of glucuronic acid (GlcA) and xylose (Xyl) (Liu S. et al., 2022). Pharmacological studies have shown that PSP exerts antidepressant (Shen et al., 2021), anti-aging (Ma et al., 2021), anticancer (Long et al., 2018) and antidiabetic (Li et al., 2020) effects. PSP can exert anti-inflammatory effects through the TLR4/Myd88/NF-κB pathway (Liu T. Y. et al., 2020). In the LPS-induced acute liver injury (SALI) murine model, PSP treatment significantly and inversely reduced serum levels of inflammatory cytokines tumor necrosis factor (TNF) - α and interleukin (IL)-6, as well as pyroptosis-associated inflammatory cytokines IL-18 and IL-1β, compared with controls (Xiao et al., 2022). PSP improves the tumor microenvironment and dose-dependently inhibits migration, invasion, and epithelial mesenchymal transition of hepatocellular carcinoma cells (Xu et al., 2023a). Long et al. (2018) demonstrated that PSP exerts anticancer effects through the TLR4/MAPK/NF-κB signaling pathway. In addition, PSP can prevent MPP-induced neurotoxicity in a dose-dependent manner, while inhibiting the production of reactive oxygen species and increasing the ratio of reduced glutathione/oxidized glutathione, resulting in antiapoptotic and antioxidant effects (Huang et al., 2021). At the same time, PSP can prevent depressive-like behavior as well as synaptic and neuronal damage by reducing ROS (Shen et al., 2021).

Different varieties of PS have different PSP content and acidic/alkaline differences. Polysaccharide content in PS and PC is higher than PK, whereas PS is dominated by neutral polysaccharides and PC comprised mainly acidic polysaccharides (Tao et al., 2023). After PS is washed, cut, dried and crushed, PSP can be extracted in three steps: removal of impurities, cell wall isolation, and polysaccharide isolation (Chen et al., 2020). Oligofructose with a polymerization degree between 5 and 10 is the main component of PSP (Singh et al., 2016). Oligofructans are oligosaccharides widely found in natural plants, which can be obtained by direct extraction or by enzyme synthesis from different substrates, and consist of linear chains of fructose units linked by β (2-1) (Sabater-Molina et al., 2009; Silva et al., 2023). Oligofructose has been shown to improve cognitive dysfunction (Sun et al., 2019), diabetes mellitus (Pengrattanachot et al., 2022), and nonalcoholic fatty liver disease (Huang et al., 2023), and is now recognized as a prebiotic that selectively promotes colonization and activity of beneficial bacteria, improves metabolism of the intestinal flora, reduces inflammation, and enhances host immunity (Huang et al., 2023). He et al. (2021) isolated two oligofructans from PC polysaccharide (PCP) with a polymerization degree between 5 and 10. Zhang J. et al. (2021) also extracted oligosaccharides from PCP.

Processing (in Chinese: Paozhi) is an important feature of Chinese medicine that originated in the early stages of its history and continues to evolve. Different herbs require different processing methods, and the effects of different processing methods on herbs are complex. Food PS are mostly processed by water or by steaming with wine to eliminate the side effect of making the mouth numb (Gan et al., 2019). Steam-processed PS has been used as a food source for thousands of years, and the Chinese Qing Dynasty medical book *Yao Pin Jian Yi* recorded that “long time steaming system is ripe, and it becomes purple black,” which believed that the original function of PS could be enhanced after steaming. The reason for this is that during the steaming process, PS can undergo acid methanolysis to hydrolyze polysaccharides, producing different types of secondary polysaccharides including oligosaccharides and monosaccharides (Bian et al., 2022). Wang et al. (Wang J. et al., 2022) also confirmed that the main components of polysaccharides in PS after steaming are Gal and GalA, and a small amount of Man, Glc, Ara, Rha, and GlcN. Repetitive cooking and drying of polysaccharides obtained from the rhizomes of PSP is considered a key step to improve their nutritional activity (Liu et al., 2024). Different steaming times result in significant changes in the structure of PSP, which have a significant modulating effect on immune function. Su et al. (2023) demonstrated that PSP with the gradual increase in steaming time can lead to a gradual increase in the number of CD4^+^/CD8^+^T-lymphocytes along with a gradual increase in the number of CD4^+^/CD8^+^T-lymphocytes.

In summary, polysaccharides of natural origin, especially those of traditional tonic Chinese medicines, exhibit a variety of pharmacological activities and have attracted the attention of drug developers. However, their mechanisms remain to be elucidated. Increasing research on intestinal microecology in recent years has determined that herbal polysaccharides can target the repair of the intestinal mucosal barrier for the treatment of multisystemic diseases (Yue et al., 2022); therefore, it is particularly important to gain a deeper understanding of PSP and its relationship with the intestinal mucosal barrier.

## 3 Intestinal mucosal barrier

The intestine is the largest digestive organ in the body and is in very close contact with the outside world; therefore, a strong barrier system has evolved in the intestine (Zhou et al., 2020). The intestinal mucosal barrier is the largest and most important barrier against the external environment and plays an important role in maintaining homeostasis (Groschwitz and Hogan, 2009). Under normal conditions, the intestinal mucosal barrier can be classified as a mechanical, chemical, immune, and biological barrier (Khoshbin and Camilleri, 2020). The mechanical (physical) barrier comprises a mucous layer, glycocalyx on intestinal epithelial cells (IECs), and cell connections involved in the physical barrier (Kayama et al., 2020),which serve to prevent harmful substances and bacteria from entering the intestinal cavity and causing inflammation (Peng et al., 2024). The mechanical barrier also provides intestinal motility and regulation of intestinal peristalsis to promote the elimination of food residues in the intestine, reducing the probability of bacteria staying in the intestinal epithelial for long periods or passing through the intestinal epithelium (Vanuytsel et al., 2023). The chemical barrier mainly comprises metabolites from the intestine and innate immune molecules, such as mucin, antimicrobial peptides, and lysozyme secreted by IECs. Mucins, especially mucin-2 (Muc-2), are important components of the skeleton of the intestinal mucus layer of the chemical barrier (Hansson and Johansson, 2010). The immune barrier mainly comprises secretory immunoglobulin A(SIgA). on the surface of the epithelial, gut-associated lymphoid tissue, and immune cells distributed in the epithelial and sub epithelial layers of the intestine. SIgA is the most abundant immunoglobulin in the intestine, and is mainly present in the upper layer of epithelial mucus. It is the main effector factor of the local immune response in the intestinal epithelium, defending against pathogen adhesion and colonization in the intestine (Peterson and Artis, 2014). The number of CD8^+^ and CD4^+^ lymphocytes in the intestinal epithelium increases upon stimulation, promoting the secretion of interleukin (IL)-6, IL-10, and interferon-α. Various cytokines produce cytotoxicity to combat pathogens, repair theintestinal barrier, and promote intestinal healing (Sarrabayrouse et al., 2015). The intestinal flora is a general term for microorganisms living in the human gut (Che et al., 2022). It is not only an important component of the biological barrier, but it also plays an important role in the functioning of other barriers and promotes the joint functioning with the intestinal mucosal barrier (Zheng et al., 2023).

### 3.1 Intestinal flora as a basis for the functioning of the intestinal mucosal barrier

The intestinal tract of healthy adults contains approximately 10^13^ to 10^14^ microorganisms with a complex structure that is influenced by factors such as genotype, diet, age, disease, and lifestyle (Adak and Khan, 2019). Under physiological conditions, the large intestinal flora remains relatively stable and constitutes a complex microecological environment. Intestinal specialized anaerobic bacteria (e.g., *Bifidobacterium bifidum*, *Lactobacillus lactis*) can defend against pathogen invasion, synthesize vitamins, produce a number of enzymes, and participate in nutrient metabolism, and can form an intestinal biobarrier through the phosphoric acid wall and interconnection with the human intestinal mucosa to prevent invasion of harmful microorganisms or toxins, which constitutes a biobarrier (Albillos et al., 2020). The tight junction (TJ) between the intestinal epithelial cells (IEC) is a major component of the mechanical barrier and is responsible for closing the intercellular space and preventing harmful substances and bacteria from entering the intestinal lumen and triggering an inflammatory response (Kaminsky et al., 2021; Wu et al., 2023). Changes in intestinal flora can lead to TJ disruption and increased intestinal permeability. Studies have shown that F/B is associated with LPS production, and a dysregulation of the ratio of the two promotes LPS production and activates the TLR4/MyD88/NF-κB pathway inducing inflammatory factors such as IL-1β, IL-6 and TNF-α to disrupt TJs and increase intestinal permeability (Martin-Subero et al., 2016; Zou et al., 2023). In the chemical barrier, mucins are secreted by cup cells and form a protective homeostatic barrier between the resident microbiota and potential immune cells in the colon (Bergstrom and Xia, 2013). In ulcerative colitis (UC) model mice, the number of goblet cells and the expression of Muc-2 are significantly reduced and is accompanied by dysregulation of the intestinal flora (Hu et al., 2021). Dysregulation of the intestinal flora leads to dysregulation of metabolites and proteins, including SCFAs, vitamins, tryptophan, and bile acids. In turn, these metabolites (predominantly SCFAs) can stimulate goblet cells to promote Muc-2 expression through the β-oxidation-like pathway-induced hypoxia-specific activation of HIF-2α (Hsieh et al., 2015; Ma et al., 2022). SIgA, the most abundant immunoglobulin in the intestine, is a major effector of the local immune response of IECs against adhesion and colonization of pathogens in the intestine (Peterson and Artis, 2014). Intestinal SIgA is closely linked to the intestinal flora, and studies have shown that germ-free mice produce a small amount of SIgA, which increases rapidly after bacterial colonization (Bos et al., 1989). Among them, *Bifidobacterium* and *Lactobacillus* promote the secretion of SIgA, and in addition, *Bifidobacterium* and *Lactobacillus* mediate the balance of Treg/Th17 in relation to this, which affects cytokine homeostasis (de Toro-Martín et al., 2017).

### 3.2 Damage to the intestinal mucosal barrier induces and exacerbates multisystemic diseases

There is growing evidence indicating that the intestinal mucosal barrier is impaired not only in digestive disorders but also in central nervous system (CNS) disorders, metabolic disorders, and in age-related diseases. The mechanisms by which the “gut-brain axis” and the “gut-liver axis” affect other organ diseases through the gut barrier and the gut microbiota have become a hot research topic.

**FIGURE 1:**
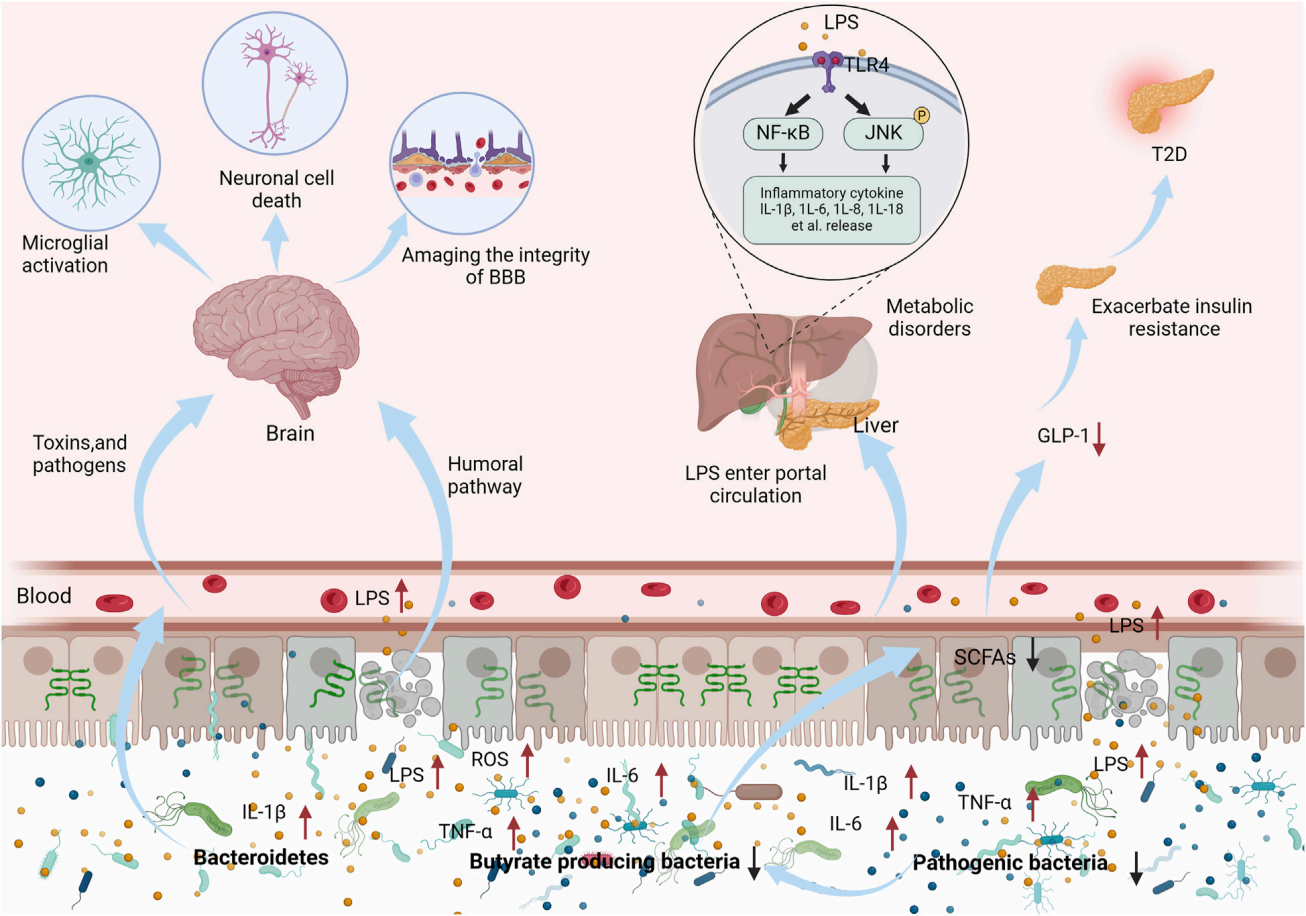
Dysbiosis of intestinal flora and reduction of SCFA can lead to damage of the intestinal mucosal barrier and entry of inflammatory factors and toxins into the bloodstream, exacerbating the development of neurological and endocrine disorders.

The intestinal mucosal barrier can be impaired in a variety of diseases. Increased translocation of bacteria, endotoxins, and other inflammatory mediators can activate relevant pathways leading to progressive exacerbation of, for example, neurodegenerative and endocrine diseases (Ghosh et al., 2021). Furthermore, aging-induced decline in intestinal mucosal barrier function can lead to the progressive exacerbation of associated diseases (Gong et al., 2023). Aging causes a significant increase in the abundance of LPS-secreting *Mycobacterium anisopliae* and a significant decrease in the abundance of butyrate-metabolizing *Bacteroides thickettsiae*, which increase inflammatory mediators and reactive oxygen species (ROS), induce TJ dysregulation, and lead to intestinal mucosal barrier damage (Wyss-Coray, 2016; Takiishi et al., 2017). Aging underlies neurodegeneration and dementia (Lechin and van der Dijs, 2009), such as AD and Parkinson’s disease (PD). Clinical studies have shown that these degenerative neuropathies are usually accompanied by damage to the intestinal mucosal barrier and increased abundance of Gram-negative bacteria, which releases LPS and circulating pro-inflammatory factors into the bloodstream, destroying the integrity of the blood-brain barrier, activating microglial cells and promoting non-programmed cell death, which further exacerbates the progression of the disease (Yang et al., 2021; Mayer et al., 2022). The function of the intestinal mucosal barrier also plays an important role in regulating metabolic diseases (e.g., obesity, T2D) (Gong et al., 2023; Zhou et al., 2023). A high-fat diet (HFD) alters the composition of the intestinal flora, leading to decreased expression of TJ protein, increased intestinal permeability, translocation of bacteria and bacterial products, and generation of systemic chronic low-grade inflammation leading to metabolic disorders (Lau et al., 2016). During T2D progression, diet-driven unfavorable microbiota composition can lead to disturbed gut flora composition with intestinal mucosal barrier damage, reduced probiotic (including butyric acid-producing bacteria) populations, increased populations of conditionally pathogenic bacteria, and increased production of pro-inflammatory LPS, which in turn exacerbate insulin resistance and metabolic disturbances in T2D via the NF-κB and the JNK signaling pathways (Lewis and Taylor, 2020; Meng et al., 2023). SCFAs can be involved in the regulation of secretion of intestinal peptides that regulate appetite and insulin secretion, such as glucagon-like peptide 1 (GLP-1) (Wang et al., 2020). The number of butyric acid-producing bacteria is also decreased in the intestinal tract of patients with T2D (Liu Q. et al., 2021) (as shown in Figure 1).

Currently, there are few therapeutic approaches specifically designed to target intestinal mucosal barrier function, and the main clinical focus is on enhancing beneficial flora or metabolites or fecal transplantation techniques (Li D. et al., 2023). Effective ingredients in TCM can repair or alleviate damage to the intestinal mucosal barrier, thus exerting the function of alleviating or treating digestive, non-gastrointestinal, and central nervous system diseases (Dong et al., 2022). Liu et al. (Liu F. Y. et al., 2021) showed that the active ingredient gastrodin in the medicinal food *Rhizoma Gastrodiae* could repair the intestinal mucosal barrier and alleviate atherosclerosis by improving the intestinal bacterial flora and restoring the TJ between IECs. Cui et al. (Cui et al., 2022) demonstrated through experimental studies that curcumin in the medicinal herb ginger treats PD by modulating the intestinal microbiota *Lactobacillaceae* and *Aerococceae*, as well as key metabolites tyrosine and dopamine. Therefore, PSP, which is also a major component of PS, can also prevent and control multisystemic diseases by repairing intestinal mucosal barrier damage.

## 4 Mechanisms of intestinal mucosal barrier repair by PSP

### 4.1 PSP regulates intestinal flora and metabolites to repair the intestinal mucosal barrier

**FIGURE 2:**
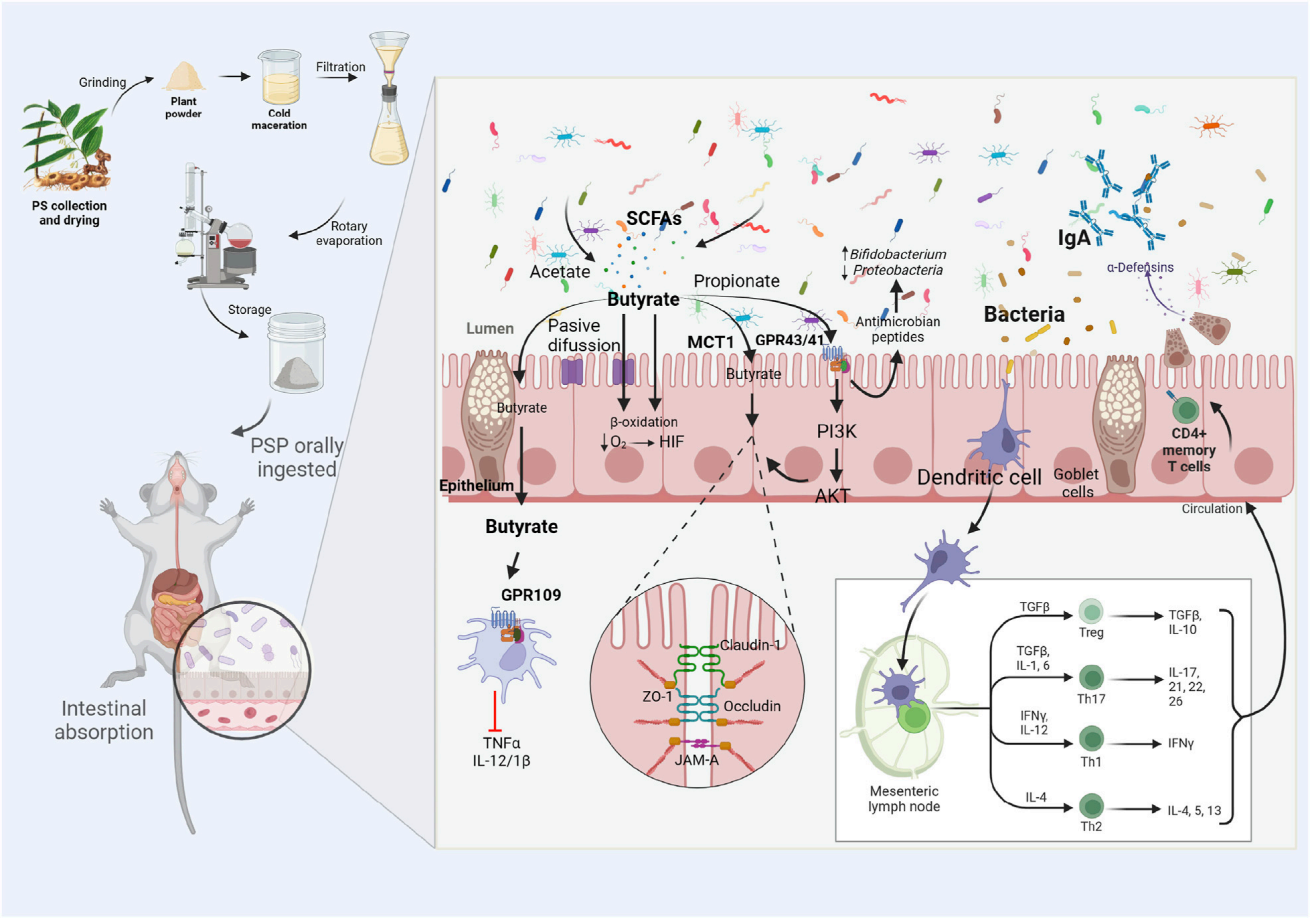
PSP acts as a prebiotic, promoting the production of probiotics and SCFAs, activating related reactions and downstream pathways, inhibiting the release of pro-inflammatory cytokines and increasing the expression of TJ proteins, thereby repairing the intestinal mucosal barrier.In addition, PSP can also repair the intestinal mucosal barrier by modulating immune cells (e.g., Th17/Treg and Th1/Th2).

As the basic backbone of PSP, oligofructose as a prebiotic can improve host metabolism by improving intestinal mucosal barrier function by altering the composition of the gut microbiota (Guarino et al., 2020; Wang et al., 2020). Prebiotic intake favors the growth of probiotics such as *Lactobacillus* and *Bifidobacterium* (Mohanty, 2018; Farias et al., 2019). PS oligofructose promotes an increase in probiotics such as *Bifidobacterium*, *Heterobacterium,* and *Alistipes* (Xu J. et al., 2023). Probiotics are defined as live microorganisms that improve health by increasing the beneficial components of the intestinal microbiota, reducing pathogen adhesion and intestinal permeability, modulating the immune response, and ensuring appropriate metabolic energy levels (Hill et al., 2014; Serek and Oleksy-Wawrzyniak, 2021). Current studies have shown that probiotics (e.g., *Lactobacillus*, *Bifidobacterium*, *Bacteroides thetaiotaomicron*, and *Akkermansia muciniphila*) are important in maintaining intestinal epithelial homeostasis and influencing intestinal barrier function (Liu Q. et al., 2020; Guarino et al., 2020). Yang et al. (2020) demonstrated that the probiotic preparations consisting of *Bifidobacterium lactis*, *Lactobacillus casei*, *B. bifidum*, and *Lactobacillus acidophilus*, significantly repaired intestinal mucosal barrier damage and inflammatory responses due to aging. Probiotics can produce SCFA through digestion, fermentation, and metabolism of dietary fiber, protein, and glycoprotein (Meng et al., 2023). Study has shown that, After entering the body,PSP promotes the proliferation of probiotic bacteria, including *Allobaculum*, *Blautia*, *Phascolarctobacterium*, *Roseburia intestinalis*, *M. anisopliae*, *Rhodococcus tumefaciens*, *Lactobacillus*, and *Prevotella*, all of which promote the production of SCFAs. *Roseburia intestinalis* metabolizes butyric acid, whereas *R. tumefaciens* and *Prevotella* metabolize isobutyric, butyric, and valeric acids (Wang et al., 2018). *In vitro* study also indicate that PSP increases the abundance of probiotics such as *Bifidobacteria* and concomitantly increases SCFAs (Xu et al., 2023c). SCFA activates G protein-coupled receptors (GPRs) 43 (FFAR2), GPR41 (FFAR3) proteins and olfactory receptor 78 (Olfr78) protein, which mediate the PI3K/Akt/mTOR pathway to enhance intestinal mucosal barrier function (Tang et al., 2022). Butyrate can also activate GPR109 protein, which in turn inhibits the release of pro-inflammatory factors such as TNF-α, IL-12, and IL-1β, affecting the health of the intestinal mucosal barrier (Dang et al., 2021). SCFAs are also important fuels for IECs, which regulate IEC function through different mechanisms in order to modulate their proliferation, differentiation, and function of subpopulations of intestinal endocrine cells, which influence intestinal motility and enhance intestinal barrier function (Martin-Gallausiaux et al., 2021). TJ injury is considered an important causal factor for intestinal mucosal barrier injury and intestinal inflammatory diseases (Zhang et al., 2023). SCFAs enhance extracellular TJ proteins (e.g., ZO-1, occludin) to reduce paracellular permeability, controlling paracellular infiltration of contents from the lumen of the intestinal tract into intestinal tissues and the somatic circulation (Xia et al., 2020; Su et al., 2022). (as shown in Figure 2)The study shows that PSP significantly increased *Akkermansia* abundance and the content of propionic acid and isovaleric acid in SCFA, and further by transplanting PSP-associated intestinal flora, it significantly increased the expression of TJ protein and decreased the content of pro-inflammatory cytokines and LPS in colon, which further confirms that PSP can repair the intestinal mucosal barrier by enhancing probiotics and increasing SCFA (Mengting, 2017).

### 4.2 PSP modulates immune cells to repair intestinal mucosal barrier

PSP has a significant effect on the regulation of the immune system.PSP promotes the expression of mucins in the jejunum as well as the expression of secretory immunoglobulin, SIgA.These results reveal that PSP improves the immune response of the intestinal mucosa.The results of the study show that PSP can improve the immune response of the intestinal mucosa (Li L. X. et al., 2022). Diagnosis of CD4^+^/CD8^+^T-lymphocytes ratio assesses the immune status,It was shown that PSP increased the ratio of CD4^+^/CD8^+^T-lymphocytes in a mouse model of cyclophosphamide-induced immunosuppression and significantly increased the splenic index and thymic index, as well as increased the expression of IL-2, IFN-γ, IgA and IgM (Su et al., 2023). During inflammatory progression, the initial CD4^+^ T lymphocytes are stimulated by antigenic signals and can differentiate into different T lymphocytes under different conditions, including Th1- and Th2-type effector cells, helper T cells 17 (Th17), and regulatory T cells (Tregs), and the balance of Th1/Th2 cells and Th17/Treg cells is important for intestinal mucosal immunity (Li L. X. et al., 2022). Th17 and Treg cells are two different subtypes of T helper cells, which play important roles in regulating the immune system. The former mainly exacerbate the inflammatory response by releasing the inflammatory factor IL-17, whereas Treg cells inhibit the activity of other immune cells by secreting anti-inflammatory cytokines, such as IL-1, IL-2, and transforming growth factor (TGF)-β, which suppresses the activity of the antigen-presenting cells and T-cell function, reducing the production of inflammatory cytokines and antibodies (Li Z. et al., 2022). Th1 and Th2 are phenotypes of host-specific immune responses mediated by CD4^+^T-lymphocytes in the pathogenesis of inflammatory diseases, and Th1/Th2 balance controls immune and inflammatory responses (Matia-Garcia et al., 2021). Th1 secretes pro-inflammatory cytokines such as IFN-γ, TNF-α, IL-1β, IL-2, IL-6, IL-8, IL-12, etc., and Th2 secretes anti-inflammatory cytokines such as IL-4, IL-10, IL-13, etc., in which IFN-γ and IL-12 secreted by Th1 inhibit the activity of Th2, and IL-4 and IL-10 secreted by Th2 inhibit the activity of Th1. Th1 activity, indicating that Th1 and Th2 can antagonize each other in pro-inflammatory/anti-inflammatory (Martino et al., 2012; Woda et al., 2016). It was shown that PSP enhanced intestinal function in aged mice by suppressing the expression of Th17 and Th1-related pro-inflammatory cytokines, including IL-6, IL-1β, and TNF-α, and increasing the expression of inflammation suppressor cytokines IL-4 and IL-10 secreted by Treg and Th2 cells in colonic tissues (Li L. X. et al., 2022). (as shown in Figure 2).

## 5 PSP repairs intestinal barrier damage in disease states to combat multisystemic diseases

### 5.1 Diseases of the nervous system

PS has been shown to improve memory (Che et al., 2022). Previous studies have confirmed that PSP prevents Aβ (Sabater-Molina et al., 2009; Liu et al., 2024) and MPP-induced neurotoxicity through the PI3K/Akt and Nrf2 signaling pathways (Zhang et al., 2015; Huang et al., 2021). AD, a central neurodegenerative disease, is the most common type of dementia, with progressive memory deficits, impaired cognitive perception of objects, marked personality changes and verbal expression problems, among other typical symptoms (Kesika et al., 2021). Studies have shown that Aβ deposits were found in the intestinal tracts of cadaver AD patients; the same was found in the intestinal tracts of APP/PS1 mice with impaired intestinal mucosal barrier and inflammation (Liu G. et al., 2023). Luo et al. (2022) isolated and purified the monomeric polysaccharide PSP-1 from PSP to improve cognitive dysfunction in 5xFAD mice by decreasing *Helicobacter pylori* abundance, increasing the abundance of probiotic bacteria, such as *Akkermansia*, rebuilding the intestinal microbiota composition, decreasing the inflammatory response and intestinal Aβ deposition, repairing the intestinal mucosal barrier and improving cognitive dysfunction in 5xFAD mice. Aging can also cause cognitive deficits that progressively worsen with age. Among them, a change in the ratio of F/B due to aging is an important cause of cognitive impairment. Liu Z., (2023) showed that PSP could alleviate cognitive impairment by restoring the F/B ratio of the intestinal flora. External stress not only triggers depression, but also leads to an imbalance in the intestinal flora, which increases intestinal permeability, providing a pathway for lumen-derived molecules, toxins (e.g., LPS), and pathogens to reach the brain parenchyma, activate local immune cells and trigger neuroinflammation, which in turn affects the onset and development of depression (Liu L. et al., 2023). Zhang (Zhang et al., 2023) and others showed that PSP remodeled the intestinal flora, increased the expression of TJ proteins ZO-1 and occludin, and reduced neuroinflammation formed by the entry of LPS and inflammatory factors into the bloodstream to alleviate depression. In addition, through transplantation of PSP-associated intestinal flora, could also increase the expression of TJ proteins, reduce LPS, and inhibit inflammatory responses in the hippocampal area, and alleviate depression, effects similar to that of direct administration of PSP (Table 2).

**TABLE 2:** Mechanisms of PSP against multisystemic diseases by repairing the intestinal mucosal barrier.

No.	Polysaccharide name	Function	Dosage	Treatment time	Administration method	Mechanisms of action	References
1	PSP-1	Alleviation of AD	30 mg/kg/day	90 days	Gavage	Decreases *Helicobacter pylori* abundance, increases probiotic abundance such as *Akkermansia*, reestablishing the composition of the gut microbiota, decreases the inflammatory response and intestinal Aβ deposition, and repairs the intestinal mucosal barrier	(Luo et al., 2022)
2	PSP	Alleviation of cognitive dysfunction in aging	150/300/600 mg/kg/day	56 days	Gavage	Restores the ratio of F/B of intestinal flora	(Liu Z., 2023)
3	PSP	Anti-depressant	400 mg/kg/day	42 days	Gavage	Reshapes intestinal flora and increases the expression of the ZO-1 and occludin TJ proteins, and reduces the neuroinflammation formed by LPS and inflammatory factors in the bloodstream	(Zhang et al., 2023)
4	PCP	Improve nutritional obesity	300/600 mg/kg/day	84 days	Gavage	Reduce LPS release, inhibit TLR4 receptor expression, increase the expression of tight junction proteins ZO-1 and Occiudin, increasing Muribaculaceae and Prevotella spp which are significantly correlated with SCFAs	(Liu Y. P., 2023)
5	PCP1	Treatment of NAFLD	200/400 mg/kg/	56 days	Gavage	Regulates the intestinal flora, decreases the relative abundance of the harmful bacteria *Helicobacter* and *Acinetobacter spp*. and increases the relative abundance of the genera *Allobaculum*, *Ruminococcus* and *Bifidobacterium*, further promoting the production of SCFAs	(Liu W. et al., 2022)
6	PSP	Treatment of T2D	—	—	—	Increases abundance of SCFA-producing bacteria such as *Prevotella_9*	(Ming-chen et al., 2021)
7	PSP	Improvement of obesity	100/200/400 mg/kg/day	56 days	Gavage	Increases the abundance of probiotics such as *Lactobacillus* and *Barnesiella*	(Zhang Z. et al., 2021)
8	PCP	Regulation of lipid metabolism disorders	120/240/480 mg/kg/day	98 days	Gavage	Increases the abundance of SCFA-producing bacteria such as *Clostridium_sensu_stricto_1*, *Roseburia*, *Bifidobacterium*, *Streptococcus*, and *Allobaculum*, increases SCFA content, repaired intestinal mucosal barrier	(Wang, 2017)
9	Polysaccharide containing tablets of PCP	Treatment of T2D	6.25/12.5/25 mg/kg/day	35 days	Gavage	Increase the abundance of probiotic *Prevotellaceae* and decrease the abundance of LPS-producing bacterium Desulfovibrionaceae, repair the intestinal mucosal barrier	(Guo, 2023)
10	PCP	Mitigation of UC	0.5/2/5 mg/kg/day	14 days	Gavage	Improves the expression of TJ proteins in the colon and promotes the increase of beneficial bacteria such as *Bifidobacterium*, Heterophyllum, and *Alistipes*	(Xu J. et al., 2023)
11	PSP-W-1	Relief from inflammatory bowel disease	100/200/400 mg/kg/day	7 days	Gavage	Repairs TJ to alleviate colitis by increasing the abundance of beneficial bacteria such as *norank_f_Muribaculaceae*, *Lactobacillus*, and *norank_f_norank_o_Clostridia UCG-014*	(Gong et al., 2024)
12	PSP-NP	Improvement of intestinal function in aged mice	50/100/200 mg/kg/day	42 days	Gavage	Regulation of Th17 and Treg cell-associated cytokines, balances the composition of intestinal flora and SCFA production, and improves inflammation and oxidative stress	(Li L. X. et al., 2022)

### 5.2 Endocrine system diseases

Intestinal mucosal barrier damage is strongly associated with the development of metabolic disorders (Chelakkot et al., 2018). Studies have shown that PS aqueous extracts can effectively reduce HFD-induced obesity and hepatic steatosis by increasing beneficial bacteria, decreasing colonic fat digestion/absorption, and increasing hepatic lipid metabolism (Ou et al., 2024). As a major component in PS, PSP also has potential in the treatment of obesity, lipid metabolism disorders and non-alcoholic fatty liver disease (NAFLD). Yapeng Liu showed that PCP can reduce LPS release, inhibit TLR4 receptor expression, reduce inflammatory response, increase the expression of TJ proteins ZO-1 and Occiudin, repair the intestinal mucosal barrier, and improve nutritional obesity by increasing *Muribaculaceae* and *Prevotella spp* which are significantly correlated with SCFAs (Liu Y. P., 2023). Liu W. et al., (2022) extracted the neutral polysaccharide PCP1 from PC. PCP1 has a protective effect against NAFLD by a mechanism that regulates the intestinal flora, decreases the relative abundance of the harmful bacteria *Helicobacter* and *Acinetobacter spp*. and increases the relative abundance of the genera *Allobaculum*, *Ruminococcus* and *Bifidobacterium*, further promoting the production of SCFAs such as isobutyric acid and isovaleric acid. Modern medicine has shown that PSP shows great potential in the prevention and treatment of endocrine diseases such as diabetes and hyperlipidemia. In China, in addition to the Han ethnic group, the ethnic groups of Tibetan, Mongolian, Miao, Yi, Qiang, and Tu also use PS for the prevention and treatment of T2D (Shi et al., 2023). PSP can affect glucose and lipid metabolism by regulating the intestinal microbiota, especially by increasing the abundance of bacteria producing SCFA (Zhang H. Y. et al., 2021). Yang et al. (Ming-chen et al., 2021) showed that the abundance of SCFA-producing bacteria *Prevotella_9* increased in T2D mice after PSP intervention. Zhang et al. (Zhang Z. et al., 2021) reported that PSP fermentation increased the abundance of probiotics such as *Lactobacillus* and *Barnesiella* in obese model mice. Wang (2017) reported that PCP improved intestinal flora structure and function, increased the abundance of SCFA-producing bacteria *Clostridium_sensu_stricto_1*, *Roseburia*, *Bifidobacterium*, *Streptococcus*, and *Allobaculum*, increased SCFA content, repaired the intestinal mucosal barrier, and alleviated lipid metabolism disorders. Xinyue Guo (Guo, 2023) developed a polysaccharide containing tablets (containing tablets weighing about 0.6 g/tablet and containing no less than 0.28 g/tablet of PCP) with PCP as raw material and relevant excipients, which can increase the abundance of probiotic *Prevotellaceae* and decrease the abundance of LPS-producing bacterium *Desulfovibrionaceae*, repair the intestinal mucosal barrier, and treat T2D.(summarized in Table 2).

### 5.3 Diseases of the digestive system

Damage to the intestinal mucosal barrier is the pathogenesis of many digestive disorders, and dysregulation of intestinal flora or metabolites can disrupt the normal immune balance in the intestinal tract and activate abnormal immune responses in the host, exacerbating intestinal inflammation and damage to mucosal tissues (Wang S. et al., 2023). PS and PSP are not traditional drugs for the treatment of digestive disorders, but have also received attention from researchers due to their role in regulating intestinal flora and metabolites to repair the intestinal mucosal barrier. Xu J. et al. (2023) found that oligofructose isolated from PCP could reduce the levels of inflammatory factors, matrix metalloproteinases, and oxidative stress in the colonic tissues of mice with UC, improve the expression of TJ proteins in the colon, and promote the increase of beneficial bacteria such as *Bifidobacterium*, *Heterophyllum*, and *Alistipes*, which could repair the intestinal mucosal barrier and thus improve UC at multiple levels. Gong et al. (2024) isolated a water-soluble polysaccharide called PSP-W-1 from steamed PS, which improve the expression of TJ proteins to alleviate colitis by increasing the abundance of beneficial bacteria *norank_f_Muribaculaceae*, *Lactobacillus*, and *norank_f_norank_o_Clostridia UCG-014*. The anti-aging effect of PSP by repairing the intestinal mucosal barrier is applicable not only to neurodegenerative diseases, but also to the treatment of intestinal disorders caused by aging. Li L. X. et al. (2022) showed that the mechanism of neutral polysaccharide of PSP (PSP-NP) in improving intestinal function in aged mice was to egulation of Th17 and Treg cell-associated cytokines, balance the composition of intestinal flora and SCFA production, and to improve inflammation and oxidative stress to repair the intestinal mucosal barrier (summarized in Table 2).

## 6 Discussion and perspectives

Entering the twenty-first century, humankind has encountered unprecedented challenges such as a century of change, the epidemic of the century, regional conflicts, and natural disasters, as well as the complex and serious global food security situation. At the social level, environmental pollution and the increasing competition in study, work, and life have caused people to be under increasing pressure, which has led to the emergence of various diseases. Therefore, it is important to find safe and effective medicines to maintain health. PS is a traditional Chinese herbal medicine and has a history of use of more than 2,000 years. Given the medicinal and food functions of PS, it has been influential in preventing diseases and performing healthcare functions. PS is starch-free, nutritionally diverse, and grows primarily in the understory of mountain forests, thickets, or along the shaded side of mountain slopes, without occupying farmland or forest land. It can not only maintain human health through “food as medicine” and “nutritional diversity”, but also improves concerns of global food shortage and promotes economic development (Si et al., 2024). In this review, we provide an overview of the properties of PSP, as the main active ingredient in PS. PSP can be used as a prebiotic to promote the increase in probiotics and their metabolites SCFAs, activate related receptors, enhance related TJs, and repair the IEC, and prevent and control multisystem diseases.

However, it should be acknowledged that current studies on the single mechanism of PSP repair of the intestinal mucosal barrier have focused mainly on the repair of the intestinal mucosal barrier by SCFA, a metabolite of the regulatory flora. In existing reports, other metabolites such as tryptophan and bile acids have achieved protective and repairing effects on the intestinal mucosal barrier (Wang S. et al., 2023); therefore, future research on the mechanism of PSP that repairs the intestinal mucosal barrier should consider whether PSP works by regulating other metabolites. In this review, we report that the intestinal flora is the basis of other barriers, and plant-based polysaccharides can also promote repair of the intestinal mucosal barrier by targeting intestinal flora and regulating the dynamic balance of Th1/Th2 and Th17/Treg (Wang X. et al., 2023). Although, there are a few reports indicating that PSP can regulate immune cell balance through intestinal flora or repair the intestinal mucosal barrier, and thus, act as a potential immune modulating drug, which could be the focus of future research. Furthermore, the extraction process, purity control, preparation method, and selection of the dosage form of PSP will also affect the efficacy of the drug. Thus, additional detailed studies and research are needed to support the translation of PSP into drugs or health products.
